# *KLF6SV1* siRNA inhibits proliferation of human lens epithelial cells

**Published:** 2012-03-03

**Authors:** Ying Su, Yixin Qu, Chenggong Jiang, Lijuan Liu, Yanchun Shan, Feng Wang

**Affiliations:** 1 Department of Ophthalmology, First clinical college of Harbin Medical University, Harbin, China; 2 Medical College, Jinan University, Guangzhou, China

## Abstract

**Purpose:**

To investigate whether transfection with Krüppel-like factor 6 splice variant 1 (*KLF6SV1*) siRNA can inhibit proliferation of human lens epithelial cell (HLEC).

**Methods:**

Plasmid containing *KLF6SV1* siRNA was used to decrease the level of KLF6SV1 protein in HLEC. The expression of protein27 kinase inhibition protein 1 (p27^kip1^) and proliferation cell nuclear antigen (PCNA) was tested with western blot. Cell proliferation was assayed by 3-(4,5-dimethylthiazolyl-2-)-2,5-diphenyltetrazoliumbromide (MTT) assay and bromodeoxyuridine (BrdU) incorporation.

**Results:**

*KLF6SV1* siRNA can decrease KLF6SV1 expression which leads to increased levels of p27^kip1^ and decreased expression of PCNA in HLEC. Cells transfected with pKLF6SV1 siRNA showed less viability compared with the control group in vitro.

**Conclusions:**

*KLF6SV1* siRNA can effectively inhibit HLEC proliferation. It can be regarded as a novel target to treat posterior capsular opacity (PCO).

## Introduction

Posterior capsule opacification (PCO) is the most common cause of visual loss after successful cataract surgery [1,2]. Nd-YAG laser posterior capsulotomy is required for restoring vision [3]. PCO arises from residual LECs at the equator and under the anterior lens capsule after cataract surgery. These cells proliferate and migrate onto the posterior capsule underlying the intraocular lens and into the light path. Many of these cells undergo epithelial to mesenchymal transition, resulting in the formation of fibroblasts and myofibroblasts, which lead to capsular opacification [4].

Kruppel-like factor 6 (*KLF6*) single nucleotide polymorphisms associated with increased prostate cancer risk increases alternative splicing of the gene into three biologically active, cytoplasmic isoforms, Kruppel-like factor 6 splice variant 1 (KLF6SV1), Kruppel-like factor 6 splice variant 2 (KLF6SV2), and Kruppel-like factor 6 splice variant (KLF6SV3), in both normal and cancerous tissues [5,6]. KLF6SV1 plays an important role in the regulation of cellular differentiation, proliferation, growth-related signal transduction, and apoptosis [7,8]. KLF6SV1 is upregulated in prostate cancer tumors compared with normal prostate tissue [9,10] and the activity of one of the splice isoforms KLF6SV1 directly opposes KLF6 effects on cell proliferation, colony formation, invasion, and in vivo tumor growth [11,12]. Our previous results confirmed that transfection with both overexpression of *KLF6* and S phase kinase-interacting protein 2 (*Skp2*) siRNA can inhibit the proliferation of rat lens epithelial cells (rLEC) [13,14].

There isn’t a previous report on the effect of KLF6SV1 on proliferation of LECs. In the present study, we examined whether transfection with *KLF6SV1* siRNA could upregulate protein27 kinase inhibition protein 1 (p27^kip1^) in human lens epithelial cell (HLEC) (SRA01/04) and their effect on HLEC proliferation in vitro with 3-(4,5-dimethylthiazolyl-2-)-2,5-diphenyltetrazoliumbromide (MTT) and bromodeoxyuridine (BrdU).

## Methods

### Reagents

KLF6sv1 was purchased from Invitrogen company (Grand Island, NY). P27 kip1 antibody and proliferation cell nuclear antigen (PCNA) was purchased from Santa cruz biotechnology Inc. (Santa Cruz, CA). Eagle’s minimal essential medium (MEM), streptomycin and penicillin were purchased from Gibco (Grand Island, NY). Fetal calf serum were purchased from Invitrogen. Hanks solution was purchased from Gibco. Poly-lysine was purchased from Sigma (St. louis, MO). Culture plates were purchased from BD Biosciences (San Jose, CA). Hybond-P polyvinylidene difluoride (PVDF) membrane was purchased from Amersham Pharmacia Biotech (Piscataway, NJ). Methylthiazolyltetrazolium (MTT) was purchased from Sigma. The MTT cell proliferation kit was purchased from the ATCC (Manassas, VA).

### Cell culture of HLEC

HLEC (SRA 01/04) were purchased from the ATCC and cultured in Eagle’s minimal essential medium (MEM) supplemented with 20% fetal bovine serum and gentamicin, 100 U/ml penicillin, and 100 μg/ml streptomycin in a six-well plate at 37 °C and 5% CO_2_ and 95% air. All experiments were performed with HLEC cells between passages 18 and 25 [15].

### KLF6SV1 siRNA plasmid construct

Vector pSuppressorNeo is a vector used to generate biologically active siRNAs from the U6promoter. Synthetic oligonucleotide primers (5′-GAT CCC CTG GCG ATG CCT CCC CCG ACt tca aga gaG TCG GGG GAG GCA TCG CCA TTT TTG GAA A-3′ and 5′-AGC TTT TCC AAA AAT GGC GAT GCC TCC CCC GAC tct ctt gaa GTC GGG GGA GGC ATC GCC AGG G-3′) were annealed and then were introduced into pSuppressor Neovector.

HLEC transfected with plasmid containing *KLF6SV1* siRNA, empty vector only, and medium only served as the experimental groups, the vehicle control group, and the blank control group, respectively. Transfection was performed in 60 mm plates using 3 µg (1 µg/µl) vector in 10µl of Metafectene Pro reagent (Biontex, Martinstried, Germany). After 48 h of transfection, cells were treated with G418 (Life Technologies, Grand Island, NY) for 2 weeks for positive clone selection. After G418 treatment, several stable transfectant cells were cloned. Each clone was screened for expression of KLF6 by western blot analysis [16].

### Western blot analysis

HLEC in lysis buffer containing 150 mM NaCl, 1 mM EDTA, 1% Nonidet P-40, 1% deoxycholate, 0.1% SDS, 20 mM Tris-HCl, pH 7.5, and protease inhibitors was crushed by ultrasonic shaft. Protein concentration was measured using absorbance spectroscopy. Protein was separated on a 10% SDS-polyacrylamide gel and transferred to nitrocellulose membranes. After blocking with 5% nonfat milk, the membranes were incubated with primary antibodies against KLF6SV1, p27 ^kip1^, and PCNA overnight at 4 °C, followed by incubation with secondary antibodies.

The membrane was then assayed using the enhanced chemiluminescent kit (ECL, Thermo Scientific, Rockford, IL) and scanned with ChemiDoc™Doc XRS+ system (Bio-Rad, Hercules, CA). The density of each band was obtained using Quantity One 4.6.2 basic software (Bio-Rad). Values were expressed as fold change relative to control and normalized to glyceraldehyde 3-phosphate dehydrogenase (GAPDH) as a loading control [16].

### 3-(4,5-dimethylthiazolyl-2-)-2,5-diphenyltetrazoliumbromide (MTT) assay

Quadruple samples of rLEC were grown on 96 well plates and were infected with 2 µg (1 µg/µl) of either of the two vectors in 10 µl of Metafectene Pro reagent, or were not infected. After 2, 4, 6, 8, and 14 days, wells were incubated in a medium containing yellow tetrazolium for 20 h [16]. Cell viability ratio=(1-A value of transfection group/A value of non-transfection group)×100%.

### Cell proliferation and bromodeoxyuridine (BrdU) incorporation

Cells (5.0×10^3^) were plated onto a 24-well multiwell plate (Falcon, Becton Dickinson, Franklin Lakes, NJ) and allowed to attach for 24 h. The culture medium was then replaced with fresh medium. Cells were trypsinized and counted using a cell counter (Coulter Z1; Coulter, FL). For BrdU incorporation, cells growing on coverslips were incubated with 10 µmol/l BrdU for 3 h. After fixing in cold methanol/acetone (1:1) for 10 min, the cells were sequentially incubated in 1.5 mol/l HCl for 10 min. Then the cells were washed with PBS and incubated with the mouse anti-BrdU primary antibody (Santa Cruz, CA) for 1 h. The cells were washed four times with PBS. Cells with different BrdU-incorporation patterns were analyzed and counted with a conventional microscope [17,18].

### Statistical analysis

The data were analyzed by the two-tailed Student *t*-test using SPSS 10.0 (IBM Inc., Beijing, China) and a p<0.05 was considered significance.

## Results

### Downregulation of *KLF6SV1* in HLEC transfected with pKLF6SV1 siRNA

**Figure 1:**
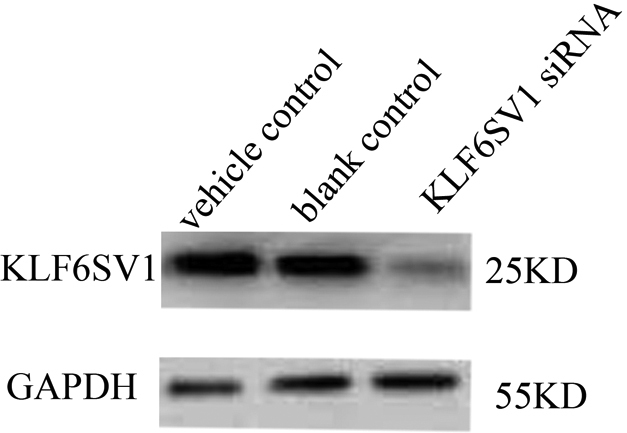
Downregulation of *KLF6SV1* in HLEC transfected with pKLF6SV1 siRNA. There is KLF6SV1 expression in the HLEC transfected with vehicle control (lane 1) or blank control (lane 2). Little expression of KLF6SV1 was detected in HLEC transfected with pKLFSV1 siRNA which indicates *KLF6SV1*siRNA can effectively decrease the expression of KLF6SV1 (lane 3).

We explored the biologic role of *KLF6-SV1* using siRNA-mediated gene silencing to specifically downregulate its expression. There is expression of KLF6SV1 protein in the HLEC transfected with vehicle (Figure 1, lane 3) or without transfection (Figure 1, lane 2). Little expression of KLF6SV1 was detected in HLEC transfected with pKLFSV1 siRNA (Figure 1, lane 3).

### Upregulation of *p27^kip1^* in HLEC transfected with *pKLF6* cDNA

**Figure 2:**
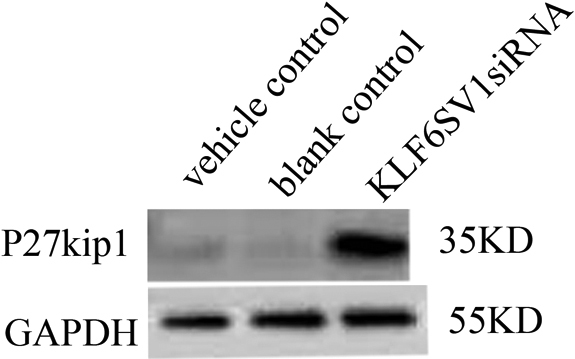
Upregulation of *p27^kip1^* in HLEC transfected with pKLF6SV1 siRNA. Expression of *p27^kip1^* increased in HLEC transfected with pKLFSV1 siRNA (lane 3) compared with vehicle control (lane 1) and blank control (lane 2).

Upregualtion of *p27^kip1^* by transfection of skp2 siRNA can inhibit the proliferation of rabbit tennon’s fibroblast cells after glaucoma surgery which indicate it is important for inhibition of cell proliferation [18]. Expression of p27^kip1^ increased in HLEC transfected with pKLFSV1 siRNA (Figure 2, lane 3) compared with vehicle control (Figure 2, lane 1) and blank control (Figure 2, lane 2). Upregulation of *p27 ^kip1^* made it possible for us to investigate the effect of *p27 ^kip1^* inhibition on the proliferation of HLEC.

### Downregulation of *PCNA* in HLEC transfected with pKLF6SV1 siRNA

**Figure 3:**
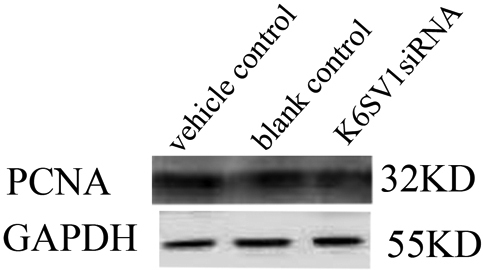
Downregulation of *PCNA* in HLEC transfected with pKLF6SV1 siRNA. Expression of *PCNA* decreased in HLEC transfected with pKLF6SV1 siRNA (lane 2) compared with blank control (lane 3) and vehicle control (lane 1).

PCNA is important marker for cell proliferation. Expression of PCNA decreased in HLEC transfected with pKLF6SV1 siRNA (Figure 3, lane 3) compared with vehicle control (Figure 3, lane 1) and blank control (Figure 3, lane 2) which indicated that pKLF6SV1 siRNA can decrease the expression of *PCNA*.

### *KLF6SV1* siRNA decrease cell viability of HLEC

#### Cell proliferation assay by BrdU

**Figure 4:**
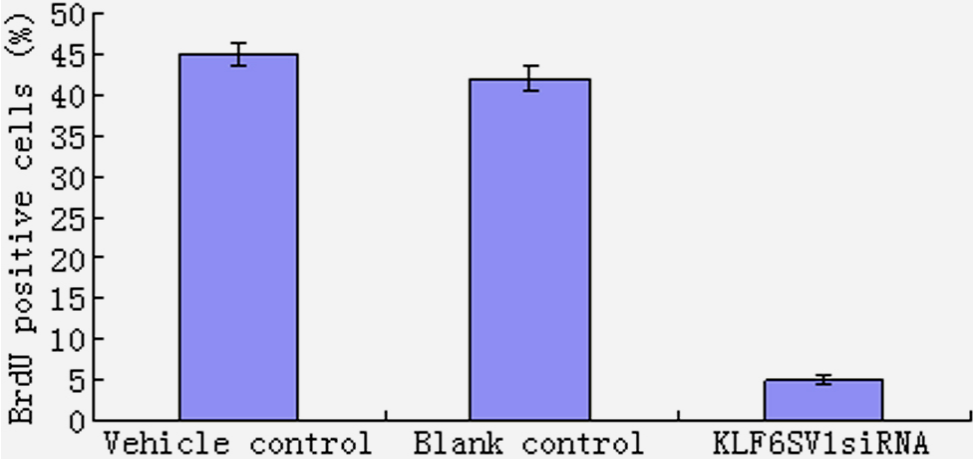
Cell proliferation assay by BrdU. Brdu positive cells decreased in pKLFSV1 siRNA transfected cells compared with HLEC of vehicle control and blank control (p<0.01 versus vehicle and blank control).

Brdu positive cells decreased in pKLFSV1 siRNA transfectant cells compared with vehicle control and blank control (p<0.01 versus vehicle and blank control, Figure 4).

#### Cell viability assay by MTT

**Figure 5:**
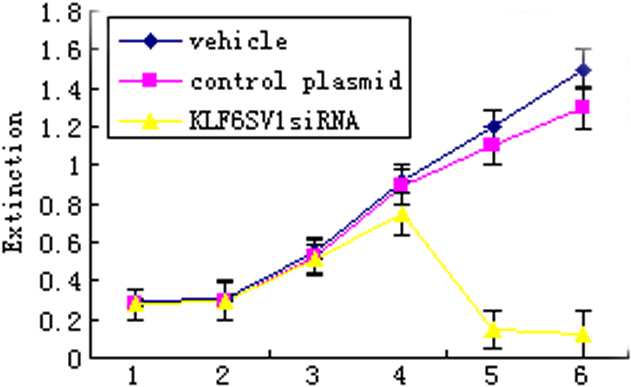
Cell viability assay by MTT. Cell viability in HLEC significantly decreased by the 5th day after transfection with pKLF6SV1 siRNA compared with vehicle control and blank control.

Cell viability in pKLF6SV1 siRNA transfected HLEC significantly decreased by the 5th day after transfection when compared with vehicle and blank control (Figure 5).

## Discussion

PCO is the most common postoperative complication of cataract surgery that causes visual loss [19]. Residual LECs at the equator and under the anterior lens capsule after surgery can proliferate and result in the formation of posterior capsular opacification [20].

KLF6-SV1 can decrease the level of cellular differentiation, development, proliferation, growth-related signal transduction, and apoptosis [21-24]. KLF6 inactivation and KLF6-SV1 overexpression have been associated not only with the progression of several human cancers but more importantly with patient survival [25-29].

Although KLF6-SV1 expression is present in both normal and cancerous tissues, the expression of this isoform is significantly upregulated in multiple cancers, including prostate cancer, glioblastoma, and lung cancer [30,31].

Expression levels of KLF6-SV1 have now been shown to have a prognostic association with lung and prostate cancers. In prostate cancer, increased expression levels of KLF6-SV1 at the time of prostatectomy were associated with a >4-year survival difference in men. Patients with high levels of KLF6-SV1 expression had a median survival of approximately 30 months versus 80 months in patients with low KLF6-SV1 expression [32]. Small interfering RNA (siRNA) has been demonstrated to be highly specific to down-regulate KLF6-SV1 expression [31,32].

Skp2 siRNA and overexpression of KLF6 can inhibit proliferation of LEC. Upregulation of *p27^kip1^*expression can inhibit the proliferation of rabbit LEC which indicates *p27^kip1^* is important for regulation of cell proliferation [16,17]. Our present study showed that transfection with pKLF6SV1 siRNA can inhibit proliferation of HLEC in vitro. In summary, this study has delineated the role of *KLF6SV1* in inhibition the proliferation of HLEC in vitro. Increased expression of *p27^kip1^* and decreased expression of *PCNA* after transfection with *KLF6SV1* siRNA suggest their important role for inhibition of cell proliferation.

Narla et al. [31] found that *KLF6SV1* siRNA can effectively enhance the apoptosis of cancer cells which indicate it can be used for treatment of cancer in the future. Furthermore, Our results also showed that We show that *KLF6SV1* siRNA inhibit the proliferation of HLEC by increasing the expression of *p27^kip1^* and decreasing *PCNA* expression in HLEC. This response is consistent with *KLF6* overexpression in rat lens epithelial cells.

In conclusion, the present work demonstrates for the first time that transfection with *KLF6SV1* siRNA can effectively inhibit proliferation of HLEC in vitro which indicates it can be novel target for treatment of PCO. Although *KLF6SV1* is a powerful way to inhibit the proliferation of HLEC, further work is needed before this approach can be used for the treatment of human disease.
